# Bioengineered tissue and cell therapy products are efficiently cryopreserved with pathogen-inactivated human platelet lysate-based solutions

**DOI:** 10.1186/s13287-023-03300-z

**Published:** 2023-04-07

**Authors:** María Martín-López, Cristina Rosell-Valle, Blanca Arribas-Arribas, Beatriz Fernández-Muñoz, Rosario Jiménez, Sonia Nogueras, Ana Belén García-Delgado, Fernando Campos, Mónica Santos-González

**Affiliations:** 1 Unidad de Producción y Reprogramación Celular (UPRC), Red Andaluza de Diseño y Traslación de Terapias Avanzadas (RAdytTA), Fundación Progreso y Salud, 41092 Seville, Spain; 2 Programa de Doctorado en Biología Molecular, Biomedicina e Investigación Clínica, Universidad de Sevilla, Seville, Spain; 3 Programa de Doctorado en Farmacia, Universidad de Sevilla, Seville, Spain; 4 Unidad de Terapia Celular, Hospital Universitario Reina Sofía, 14004 Córdoba, Spain; 5 Instituto Maimónides de Investigación Biomédica de Córdoba (IMIBIC), 14004 Córdoba, Spain; 6 Departamento de Farmacia y Tecnología Farmacéutica, Facultad de Farmacia, Universidad de Sevilla, Seville, Spain; 7 Tissue Engineering Group, Department of Histology, Universidad de Granada, Granada, Spain; 8 Instituto de Investigación Biosanitaria Ibs.Granada, Granada, Spain; 9 Centro de Transfusiones, Tejidos y Células de Sevilla (CTTS), Fundación Pública Andaluza para la Gestión de la Investigación en Salud en Sevilla (FISEVI), 41013 Seville, Spain

**Keywords:** Artificial tissue, Cryopreservation solution, hPL, ATMP, Stem cell therapy, Freezing

## Abstract

**Background:**

There remains much interest in improving cryopreservation techniques for advanced therapy medicinal products (ATMPs). Recently, human platelet lysate (hPL) has emerged as a promising candidate to replace fetal bovine serum (FBS) as a xeno-free culture supplement for the expansion of human cell therapy products. Whether hPL can also substitute for FBS in cryopreservation procedures remains poorly studied. Here, we evaluated several cryoprotective formulations based on a proprietary hPL for the cryopreservation of bioengineered tissues and cell therapy products.

**Methods:**

We tested different xenogeneic-free, pathogen-inactivated hPL (ihPL)- and non-inactivated-based formulations for cryopreserving bioengineered tissue (cellularized nanostructured fibrin agarose hydrogels (NFAHs)) and common cell therapy products including bone marrow-derived mesenchymal stromal cells (BM-MSCs), human dermal fibroblasts (FBs) and neural stem cells (NSCs). To assess the tissue and cellular properties post-thaw of NFAHs, we analyzed their cell viability, identity and structural and biomechanical properties. Also, we evaluated cell viability, recovery and identity post-thaw in cryopreserved cells. Further properties like immunomodulation, apoptosis and cell proliferation were assessed in certain cell types. Additionally, we examined the stability of the formulated solutions. The formulations are under a bidding process with MD Bioproducts (Zurich, Switzerland) and are proprietary.

**Results:**

Amongst the tissue-specific solutions, Ti5 (low-DMSO and ihPL-based) preserved the viability and the phenotype of embedded cells in NFAHs and preserved the matrix integrity and biomechanical properties similar to those of the standard cryopreservation solution (70% DMEM + 20% FBS + 10% DMSO). All solutions were stable at − 20 °C for at least 3 months. Regarding cell-specific solutions, CeA maintained the viability of all cell types > 80%, preserved the immunomodulatory properties of BM-MSCs and promoted good recovery post-thaw. Besides, both tested solutions were stable at − 20 °C for 18 months. Finally, we established that there is a 3-h window in which thawed NFAHs and FBs maintain optimum viability immersed in the formulated solutions and at least 2 h for BM-MSCs.

**Conclusions:**

Our results show that pathogen-inactivated solutions Ti5 allocated for bioengineered tissues and CeA allocated for cells are efficient and safe candidates to cryopreserve ATMPs and offer a xenogeneic-free and low-DMSO alternative to commercially available cryoprotective solutions.

**Supplementary Information:**

The online version contains supplementary material available at 10.1186/s13287-023-03300-z.

## Background

Cryopreservation is a cornerstone of regenerative medicine and advanced therapies, as it allows for the safe and secure transition of advanced therapy medicinal products (ATMPs) from manufacturing to final treatments for patients. This is particularly relevant for bioengineered tissues, given their immediate necessity (e.g., for burn patients) and the time-consuming procedures their manufacturing generally entails. Despite the growing number of studies using cryopreservation for ATMPs [1, 2], there is still much interest in improving this key method.

The successful cryopreservation of tissue and cells necessitates the use of cryoprotective agents (CPAs), which must preserve cellular viability, functionality and structural and biomechanical properties of tissue during the processes of freezing and thawing. Human platelet lysate (hPL) has emerged as a promising candidate to replace the use of xenogeneic sera such as fetal bovine serum (FBS) in production processes [3, 4], principally because the use of FBS might lead to the transmission of diseases of animal origin or might trigger rejection responses [5]. In the same vein, hPL could potentially substitute for FBS during cryopreservation, and some studies have suggested that hPL, together with other components, could effectively aid in the cryopreservation process [6, 7]. Dimethyl sulfoxide (DMSO) is the most widely used CPA, but it is known to trigger adverse and toxic reactions in patients including heart rate reduction and cell membrane damage after infusion of cells [8]. It is therefore necessary to reduce its concentration during the cryopreservation process.

Several xeno-free cryopreservation solutions are available in the market; however, to date, none of them base their formulation on pathogen-inactivated hPL (ihPL), which has been successfully tested as a xeno-free culture supplement for the expansion of different cell types [3]. Moreover, to comply with the latest recommendations on blood derivatives [9] that advise pathogen-reduction treatments to minimize the risk of viral and bacterial transmission in blood products [5], we have manufactured ihPL preparations to formulate our solutions [10].

In the present study, we assessed the effectiveness of xeno-free, in-house manufactured hPL and ihPL-based cryoprotectants with a low DMSO concentration for ATMPs, in an attempt to minimize the potential adverse effects on patients.

## Methods

### Study design

We formulated several xeno-free hPL-based cryopreservation solutions and evaluated their efficacy for cryopreserving ATMPs, including bioengineered tissue. For the latter, we tested cellularized NFAHs, currently used at the Unidad de Producción y Reprogramación Celular (UPRC, Seville, Spain) to treat burns. We also tested the following cell types commonly used in cell therapy: bone marrow-derived mesenchymal stem cells (BM-MSCs), human dermal fibroblasts (FBs) and neural stem cells (NSCs). ihPL and low-DMSO formulations were included in some of the solutions with the objective of providing additional safety and security. The study was performed in two Good Manufacturing Practice (GMP)-grade laboratories that manufacture and cryopreserve ATMPs for clinical use. Each GMP laboratory followed their standard methods based on previous experience (e.g., standard cryopreservation solution and number of cells) for each tissue/cell type for comparative analysis.

### Generation of cellularized nanostructured fibrin agarose hydrogels

NFAHs are artificial tissues that are used for diverse clinical applications depending on the encapsulated cell type. For the present study, the NFAH contained FBs and was generated as previously described [11]. Briefly, 4.16 ml of human plasma obtained from healthy blood donors was added to 7.5 × 10^5^ FBs resuspended in Dulbecco's Modified Eagle´s Medium (DMEM; Sigma-Aldrich, St. Louis, MO) supplemented with gentamicin (20 µg/ml) (Normon, Madrid, Spain) and 83 µl of tranexamic acid (Amchafibrin® 500 mg; Rottapharm, Milan, Italy), as an anti-fibrinolytic agent. We then added 0.25 ml of 2.2% type VII-agarose (Sigma-Aldrich) in PBS (Sigma-Aldrich). When the temperature of the solution fell to < 40 °C, 300 µl of 10% calcium chloride (B.Braun, Melsungen, Germany) was added to support the fibrin polymerization reaction. Aliquots (5 ml) were placed in 24-mm diameter Transwells (Corning, Corning, CA) and allowed to solidify at 37 °C for 2 h. Subsequently, the wells were filled with DMEM (Sigma-Aldrich) supplemented with 5% hPL and 2 U/ml heparin (Rovi, Madrid, Spain) to prevent unwanted clotting. Fibrin agarose hydrogels were kept at 37 °C for 12 days.

**Fig. 1:**
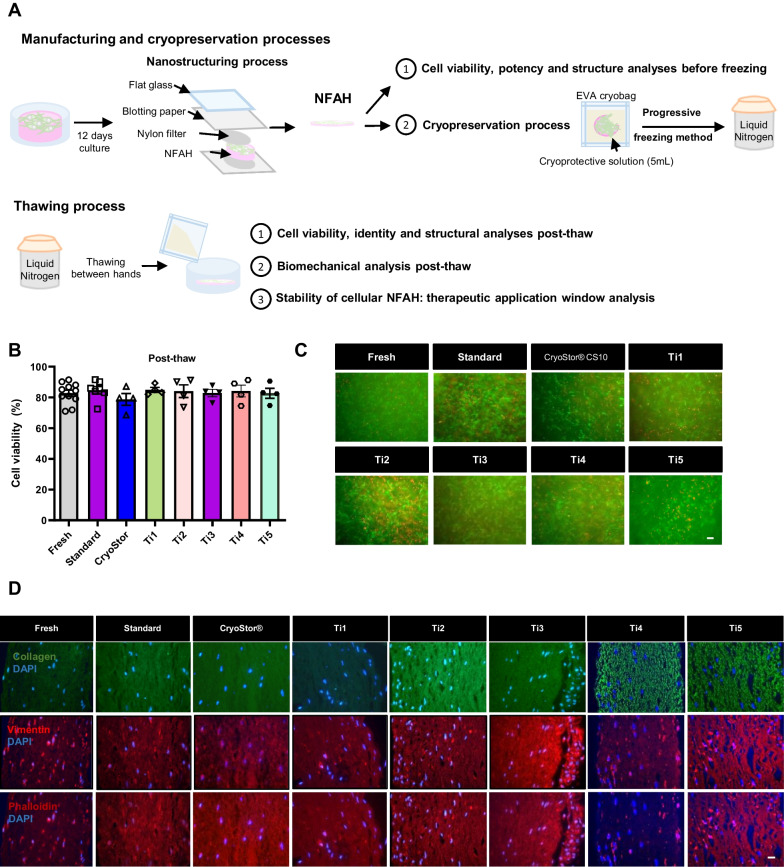
Analysis of cryopreservation of bioengineered tissues. **A** Schematic representation of the manufacturing, cryopreservation and thawing processes of cellularized nanostructured fibrin-agarose hydrogels (NFAHs). Three or four samples per group were cryopreserved with five different human platelet lysate-based formulations and compared with a control solution (70% DMEM 20% FBS) and with the commercial product Cryostor® CS10. **B** Cell viability post-thaw. **C** LIVE/DEAD® Assay staining of NFAH (scale bar: 100 µm). **D** Immunocytochemistry analysis of collagen type I, vimentin and phalloidin (scale bar: 10 µm)

We then applied the nanostructuring technique, as described [11] (Fig. 1A). Briefly, we prepared an extra‐thick western blotting filter paper (ThermoFisher Scientific, Waltham, MA) with a 10‐μm nylon net filter on top (Merck Millipore, Burlington, MA) to prevent adherence, and the hydrogel was placed over the filter. Then, another nylon net filter/blotting paper was sandwiched over the hydrogel. A flat glass weighting 0.25 kg was then quickly positioned on top for 1 min and 40 s for compression. Artificial tissues were manufactured at the UPRC (Seville, Spain).

### Cell lines

BM-MSCs were cultured and cryopreserved at the Unidad de Terapia Celular, Hospital Universitario Reina Sofía, (Córdoba, Spain) in alpha-minimum essential medium (αMEM; Lonza, Milan, Italy) supplemented with 13% (v/v) FBS (Gibco/Invitrogen, Carlsbad, CA), 2 mM ultraglutamine 1 (Lonza) and 1 ng/ml fibroblast growth factor (Miltenyi Biotec, Bergisch Gladbach, Germany).

FBs were cultured at the UPRC (Seville, Spain) in DMEM supplemented with 10% (v/v) FBS (Sigma-Aldrich), 0.1 mM non-essential amino acids (NEAA; Sigma-Aldrich), 2 mM Glutamax (Gibco/Invitrogen) and 100 μg/ml gentamicin (Sigma-Aldrich).

NSCs [12] were cultured at the UPRC (Seville, Spain) in DMEM-F12 (ThermoFisher Scientific), 0.1 mM NEAA, 100 IU penicillin/100 μg/ml streptomycin (Sigma-Aldrich), 2 μg/ml heparin (Rovi), 1% N2, 1 × B27 (both from ThermoFisher Scientific), 20 ng/ml FGF (Miltenyi Biotec), 20 ng/ml epidermal growth factor (Peprotech, Pcky Hill, NJ) and 10 ng/ml leukemia inhibitory factor (Miltenyi Biotec).

### Testing hPL-based solutions

Based on a bibliographic study and our laboratory experience, we developed different formulations to evaluate cryopreserving of NFAHs and commonly used cells. hPL and ihPL were produced as described [10].

Five different hPL-based cryopreservation solutions (Ti1, Ti2, Ti3, Ti4 and Ti5) were developed and tested for cryopreserving NFAHs and were compared against two control solutions (the standard cryopreservation solution at our laboratory and a commercially available one). The conditions and reagents used for the bioengineered tissue cryopreservation process are described in Table 1 and Additional file 1. Control 1 was selected as the gold standard solution used to cryopreserve NFAHs in accordance with previously published results of the group [13]. All cryoprotective solutions were kept on ice until use. NFAHs were placed into a Maco Biotech Freezing EVA bag (Macopharma Biotech, Tourcoing, France) containing 5 ml of cryoprotective solution, which was then sealed with a heat sealer (Seal Kit 235, Texas Technologies, TX). Subsequently, the samples were transferred to an ice pan before continuing with the freezing method (Fig. 1A and Additional file 1).

**Table 1:** Description of conditions and reagents used for bioengineered tissue cryopreservation

Cryoprotective solutions	
**Control 1**	70% DMEM + 20% FBS + 10% DMSO
**Control 2**	Cryostor® CS10
**hPL-based**	
**10% DMSO**	**Solution Ti1**
**10% DMSO**	**Solution Ti2**
**5% DMSO**	**Solution Ti3**
**ihPL-based**	
**10% DMSO**	**Solution Ti4**
**5% DMSO**	**Solution Ti5**
Freezing method	Freezing EVA cryobags^a^
Time in liquid N2 (days)	7
GMP laboratory	UPRC Seville

*DMSO* dimethyl sulfoxide, *hPL* human platelet lysate, *ihPL* inactivated human platelet lysate, *DMEM* Dulbecco's Modified Eagle’s Medium, *FBS* fetal bovine serum, *EVA* ethylene–vinyl acetate, *UPRC* Unidad de Producción y Reprogramación Celular

^a^See Additional file 1

Two cryopreservation formulations, termed CeA and CeB, were assessed for cell cryopreservation. The formulations are different to those used in tissue cryopreservation and are both based on ihPL with a reduced DMSO content (5%). For NSC cryopreservation we only assessed CeA, as it provided better results when tested on the other cell types (see results). Detailed information about the cryopreservation solutions and conditions used in each cell type and GMP facility is described in Table 2 and Additional file 1. Cryoprotective solutions were freshly made and kept on ice until use.

**Table 2:** Description of conditions and reagents used for cell cryopreservation

	Cell type		
	BM-MSCs	FBs	NSCs
**Cryoprotective solution**			
**Control 1 (standard)**	4.5% HSA + 10% DMSO	CryoStor® CS10	90%FBS + 10% DMSO
**Control 2**	Not applicable	SCB	CryoStor® CS10
**Control 3**	Not applicable	Not applicable	SCB
**ihPL-based 5% DMSO**	**Solution CeA**	**Solution CeA**	**Solution CeA**
	**Solution CeB**	**Solution CeB**	Not applicable
**No. of cells**	10 × 10^6^	1 × 10^6^ // 10 × 10^6^	0.5 × 10^6^
**Freezing method**	Nunc™ cryovials	Nunc™ cryovials	Nunc™ cryovials
	Controlled-rate freezer^a^	Mr. Frosty	Mr. Frosty
**Time in liquid N**_**2**_** (months)**	3	9	3
**Thawing method**	Water bath (37 °C)	Water bath (37 °C)	Water bath (37 °C)
GMP laboratory	HURS Cordoba	UPRC Seville	UPRC Seville

*BM-MSCs* bone marrow-derived mesenchymal stromal cells, *FBs* human dermal fibroblasts, *NSCs* neural stem cells, *DMSO* dimethyl sulfoxide, *SCB* STEM-CELLBANKER® DMSO FREE-GMP Grade, *ihPL* inactivated human platelet lysate, *HSA* human serum albumin human, *HURS* Hospital Universitario Reina Sofía, *UPRC* Unidad de Producción y Reprogramación Celular

^a^See Additional file 1

### Cell viability analysis

We determined the viability of cells embedded in the NFAH before and after cryopreservation using the LIVE/DEAD® Viability/Cytotoxicity Kit for mammalian cells (Molecular Probes, Invitrogen, ThermoFisher Scientific). We adapted the manufacturer's protocol by additionally staining cell nuclei with Hoechst solution (Miltenyi Biotec) to aid in cell counting. Cryopreserved NFAHs were thawed by hand (25.5–32 °C) and washed three times with PBS in a Petri dish. Subsequently, cells were incubated with the staining solution for 30 min at room temperature (RT) and then washed three times in PBS. The number of live and dead cells was assessed by fluorescence microscopy, counting three random fields for each NFAH sample.

For cell cryopreservation, we measured viability using trypan blue (Sigma-Aldrich) exclusion before and after thawing BM-MSCs, FBs and NSCs. Cell recovery was calculated according to the following equation: cell recovery post-thaw (%) = (alive cells × 100)/cryopreserved cells. For BM-MSCs and FBs, we additionally evaluated 24 h-recovery as follows: the thawed cells were seeded and cultured at 37 °C and 5% CO_2_ for 24 h; cells were then detached with CTS™ TrypLE™ Select Enzyme (A1285901, ThermoFisher Scientific) and counted again. Cell recovery percentage 24-h post-thaw was determined with the following equation: 24-h post-thaw recovery (%) = (alive cells at 24 h × 100) / seeded cells. We performed two cell counts per sample.

### Immunofluorescence

NFAHs were examined before and after cryopreservation. NFAHs were fixed in 3.7% buffered formaldehyde for 15 min at RT and were then dehydrated and embedded in paraffin. For phenotypic analysis, we deparaffinized and rehydrated the NFAH sections and applied heat-induced epitope retrieval with citrate buffer, pH 6 (Alfa Aesar, Haverhill, MA). Subsequently, we permeabilized and blocked the samples with 0.1% Triton X-100 (Sigma-Aldrich) and 5% donkey serum (Sigma-Aldrich) in PBS. Samples were incubated overnight with primary antibodies at 4 °C and then washed twice in PBS and once again in 0.1% Triton X-100 in PBS for 10 min at RT. Finally, we incubated the samples with secondary antibodies for 1 h at RT (see Additional file 2). All antibodies were diluted in PBS with 2% donkey serum. Nuclei were stained with Hoechst (1:100) and mounted with ProLong™ Gold Antifade Mountant (ThermoFisher Scientific). Analysis was performed using a Nikon TiS microscope (Nikon Instruments, Amsterdam, The Netherlands).

### Structural analysis

NFAH sections were stained with hematoxylin and eosin as described [14, 15] to determine their integrity (interfibrillar space). The preservation degree was determined by the quantification of the spaces produced in the fibrin-agarose fibrillar mesh by ice crystals. Empty areas were quantified with ImageJ software [16] by examining six random fields per NFAH.

### Biomechanical analysis

Evaluation of the biomechanical properties of the NFAHs was performed as described [17]. Briefly, all samples were subjected to tensile tests using an electromechanical material testing instrument (Model 3345-K3327; Instron Ltd., High Wycombe, UK). Samples were sectioned to a regular rectangular shape, oriented with their length along the direction of tension and clamped at each end. A constant distance of 1 cm between the clamps was set. Trials were run at a constant strain rate of 5 mm per min at RT. The following parameters were measured using a 50-N Instron load cell to obtain data for stress–strain curves: Young’s modulus, which characterizes the behavior of elastic material when a force is applied lengthwise, was calculated as the tangent modulus of the initial, linear portion of the stress–strain curve of each experimental run; stress at fracture break, determined by selecting the point of the stress–strain curve where the fracture occurred; and traction/deformation percentage.

### Phenotype assessment

Cells were characterized by flow cytometry before and after cryopreservation. NSCs were characterized one passage after thawing. Additional file 3 shows the full list of antibodies used for flow cytometry analysis. Live cells were suspended in PBS and incubated with conjugated antibodies for 30 min at 4 °C in the dark. To test for phenotypic robustness, we first fixed the cells with 3.7% formaldehyde (Sigma-Adrich) for 15 min at RT; they were then blocked and permeabilized with 3% bovine serum albumin (BSA; Sigma-Adrich) and 0.1% Triton X-100 (Sigma-Adrich) in PBS 30 min, RT. Cells were incubated with the antibody for 30 min at 37 °C in the dark. Fluorescence was estimated with a MACS Quant flow cytometer (Miltenyi Biotec) and results were analyzed with MACS Quantify 2.10 software. Isotype controls were run in parallel. The sample size was 10,000 cells per measurement.

### Apoptosis and proliferation

We analyzed apoptosis on FBs before and after cryopreservation using the Annexin-V-FITC Apoptosis Detection Kit (Miltenyi Biotec), as previously described [18]. Data were analyzed using MacsQuantify 2.10 software. At least 10,000 events were analyzed per sample.

NSCs were seeded and expanded for 1 week (one passage after thawing). Cells were then counted, and the population doubling (PD) was calculated according to the equation: PD = (log *y* − log *x*)/ log2, where “*y*” is the number of cells at the end of the cultivation period and “*x*” the number of cells at the beginning.

### Functional analysis of immunomodulation

Peripheral blood mononuclear cells (PBMCs) were isolated from the blood of healthy donors collected in heparin tubes. We diluted whole blood 1:1 with Roswell Park Memorial Institute (RPMI) medium (Lonza, Basilea, Sweeden) and layered 2 ml on top of 4 ml of Ficoll solution (Alere Technologies AS, Oslo, Norway) in 15-ml conical tubes. Tubes were centrifuged at 1500 g for 20 min at 20 °C, and the white layer was transferred to a clean tube and washed twice in PBS. PBMCs were stained with 5 µM carboxyfluorescein succinimidyl ester (CFSE; Sigma-Aldrich) for 10 min at RT. Cells were then washed twice with PBS and resuspended in RPMI medium.

We seeded 100,000 BM-MSCs per well into a 6-well plate and, 24 h later, we placed a 0.4 µm-diameter Transwell insert (CellStar, Kaysville UT) over each well and seeded 500,000 PBMCs per insert. PBMCs were activated with 1% phytohemagglutinin (Gibco) 24 h later. The co-culture was maintained for 7 days and PBMC proliferation was assessed by flow cytometry of CFSE expression. To assess T-lymphocyte proliferation, we also stained PBMCs with an antibody to CD3-APC to select the T-lymphocyte population and, again, measured CFSE to detect proliferative T-lymphocytes. Results were analyzed with MACS Quantify 2.10 software.

### Stability analysis

On manufacturing day (*t* = 0) and after a 3-month storage (*t* = 3) period at − 20 °C, we assessed the stability of ihPL-based solutions allocated for bioengineered tissue cryopreservation. We measured total protein, albumin and immunoglobulin G (IgG) concentrations with a Dimension System platform (Siemens Healthcare, Forchheim, Germany) and pH with a pH-meter (HI 221; HANNA Instruments, Woonsocket, RI). IgG content was assessed as a complementary measurement. Given that albumin is one of the most abundant proteins in hPL, we measured lower concentration proteins such as IgGs, which would serve as an indication of how other proteins may have degraded. The same parameters were measured for freshly made (*t* = 0), 12 (*t* = 12) and 18-month (*t* = 18) stored solutions (− 20 °C) aimed for cell cryopreservation. Furthermore, we evaluated cell solutions to determine an expiry date. With this aim, we cryopreserved FBs (10^6^ cells/ml) with 12- and 18-month stored solutions and with CryoStor® CS10, used as a control.

### Statistical analysis

Statistical analysis was conducted with GraphPad Prism, version 8 (GraphPad Software, Inc., La Jolla, CA). To assess normal distribution of data, we used the Shapiro–Wilk test. Levene’s test was used to analyze the assumption of homogeneity of variance. We applied one-way and two-way (for cell density effect assessment) analysis of variance (ANOVA) followed by Tukey’s multiple-comparisons test. Correlation analysis was performed computing the value of the Pearson correlation coefficient, *r*. Data are expressed as mean and ± standard error of the mean (SEM). *P* < 0.05 was considered as significant.

## Results

In an effort to optimize the cryopreservation of artificial tissues and cells, we formulated several xeno-free, hPL-based proprietary solutions for the cryopreservation of NFAH, a bioengineered tissue, and for common cell therapy agents produced in our GMP laboratories. Pathogen-inactivated hPL and low-DMSO concentration formulations were evaluated with the aim of offering additional safety and security.

### Bioengineered tissue

#### Formulated hPL-based cryoprotective solutions preserve cell viability and functionality and sustain matrix integrity

NFAH can be used for different clinical applications depending on the embedded cell type [11, 19–22]. The NFAH used in the present study contained FBs, thus acting as an artificial dermis.

We first examined whether Ti1, Ti2, Ti3, Ti4 and Ti5 solutions (Table 1) preserved the biological and biomechanical properties of the NFAH after one week of cryopreservation (Fig. 1A). We found that cell viability was similar (~ 80%) between fresh and cryopreserved NFAH immediately after thawing (Fig. 1B, C). Likewise, immunofluorescence analysis revealed a similar expression of collagen type I, vimentin and phalloidin (Fig. 1D), indicating that none of the solutions affected morphology or extracellular matrix production of the embedded FBs.

**Fig. 2:**
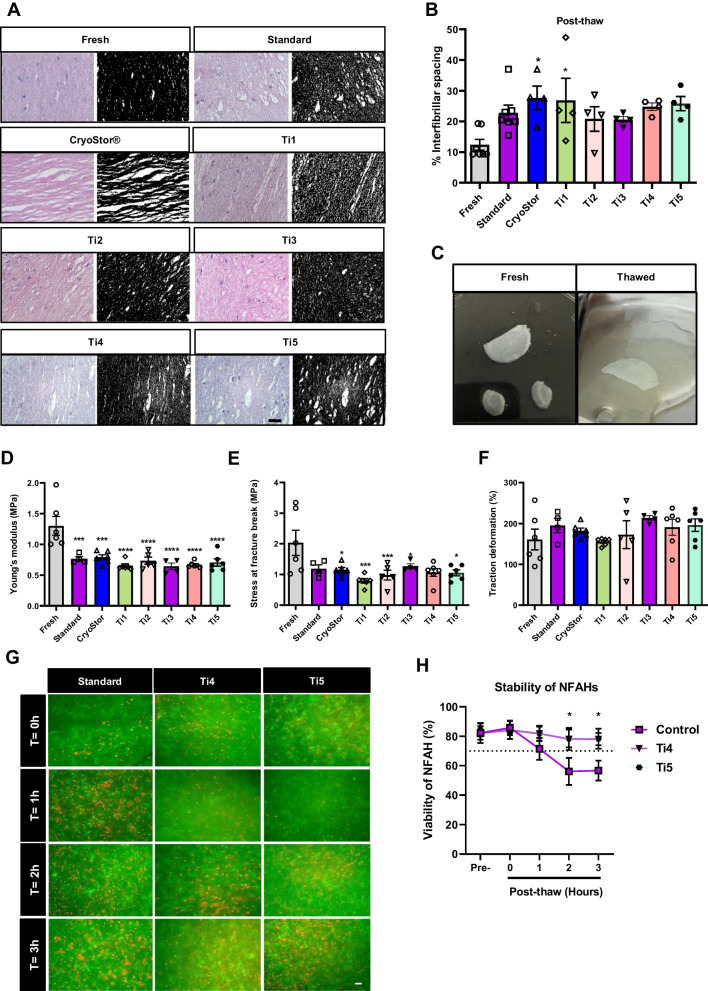
Histology, biomechanical properties and stability analyses of cryopreserved bioengineered tissues. Histological analysis: **A** fresh and thawed tissue sections for each condition were stained with hematoxylin/eosin (left) and their pictures were transformed into binary images (right) using ImageJ program for quantification of pore size (n = 6 random fields; scale bar: 50 µm); fresh fibrin-agarose mesh contrast with thawed tissue, which presents more and larger-sized pores and **B** bar graph representation of the percentage of interfibrillar spacing. Macroscopic images of NFAH: **C** Representative fresh (left) and thawed (right) NFAH show the tissue remains in a good macroscopic status after thawing. NFAH is sectioned in three parts, one halve is cryopreserved and two quarters are used for quality controls. Biomechanical properties analysis: **D** Young’s modulus, **E** stress at fracture break and **F** traction-deformation. Cell stability analysis: **G** LIVE/DEAD® Assay staining of the tissue **H** cell viability at different time points (scale bar: 100 µm). Live cells are shown in green and dead cells in red. Results are presented as mean ± SEM. One-way ANOVA differences *P < 0.05; ***P < 0.001; **** P < 0.0001

Analysis of matrix integrity (interfibrillar space) revealed an effect of solutions (F_(7,30)_ = 2.797, *P* < 0.05). While the integrity of the NFAH structure was affected irrespective of the cryoprotectant tested, the results were similar between NFAHs cryopreserved with Ti2, Ti3, Ti4 and Ti5 and the standard cryopreservation solution (Tukey’s test: *P* > 0.05, Fig. 2A, B). Figure 2C shows the aspect of representative fresh and thawed NFAH, indicating the preservation status of the tissue is macroscopically optimum.

Notably, we observed that NFAHs cryopreserved with Ti3 took significantly longer to thaw (one-way ANOVA: *F*_(6,24)_ = 7.222, *P* < 0.0001, Tukey’s test: *P* < 0.05, see Additional file 4A), although no correlation was found between thawing time and interfibrillar space (Pearson’s correlation: *P* > 0.05, see Additional file 4B).

We next tested whether the mechanical properties of NFAHs were preserved after cryopreservation. Results showed an influence of cryopreservation on Young’s modulus (one-way ANOVA: *F*_(7,35)_ = 9.402, *P* < 0.0001, Fig. 2D) independently of the solution when compared with fresh tissue. The stress at fracture break was similarly affected by cryopreservation (one-way ANOVA: *F*_(7,35)_ = 4.028, *P* < 0.001, Fig. 2E), whereas the traction deformation results were not significantly different between NFAHs cryopreserved with the different cryoprotective solutions (one-way ANOVA: *F*_(7,35)_ = 4.028, *P* > 0.05, Fig. 2F).

Overall, we did not observe significant differences in the parameters analyzed between the proprietary formulations and the standard solutions. Given that the Ti4 and Ti5 solutions are based on ihPL and are likely to be safer for clinical use, we used these for further analyses. We next explored for how long NFAHs retain their properties after thawing when cryopreserved with Ti4 and Ti5, to determine the stability of NFAHs in a clinical scenario where tissue application could be delayed. We measured the cell viability of NFAHs immediately at thaw and at 1, 2 and 3 h after thawing. NFAHs cryopreserved with Ti4 and Ti5 showed significantly greater cell stability at 3 h after thawing than equivalent NFAHs cryopreserved with the standard (Control 1) (two-way ANOVA: *time* effect: *F*_(4,40)_ = 3.230, *P* < 0.05; *solution* effect: *F*_(2,40)_ = 4.557, *P* < 0.05, Tukey’s test: *P* < 0.05, Fig. 2G, H). As both Ti4 and Ti5 gave very similar results, we conclude that solution Ti5 has the most adequate formulation to cryopreserve artificial tissues owing to its reduced DMSO concentration.

### Cells

To optimize the cryopreservation of cells, we evaluated two solutions: CeA and CeB (Table 2). Both are based on ihPL and have a low concentration (5%) of DMSO. The two formulations were selected based on previous proof of concept results (data not shown) and are different to those used for tissue cryopreservation.

#### Solution CeA maintains BM-MSC viability > 85%, has no negative effects on cell recovery post-thaw or after 24-h culture and preserves cell immunomodulatory properties

**Fig. 3:**
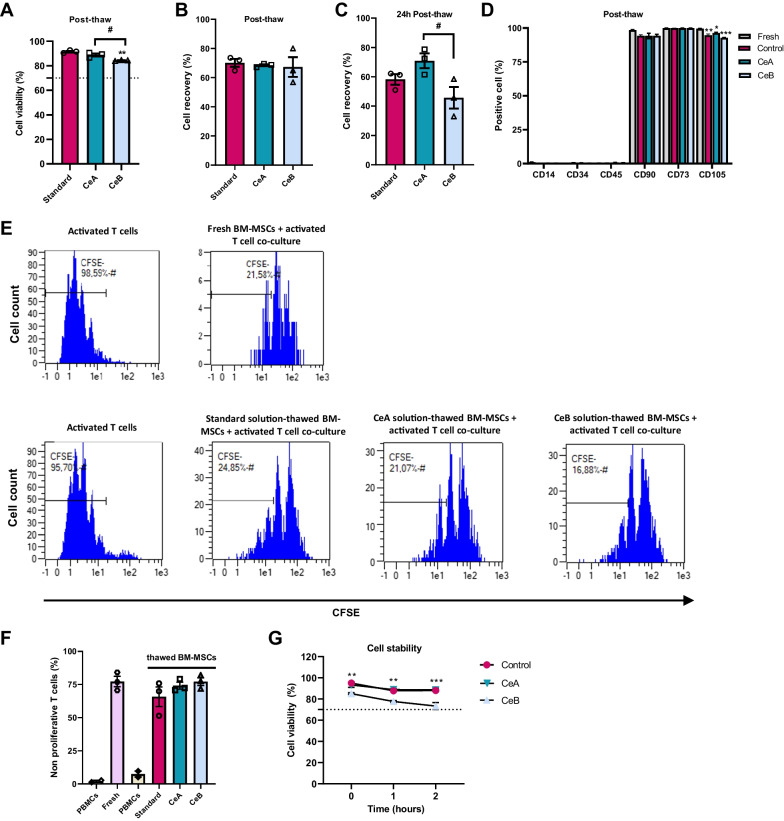
Analysis of cryopreserved bone marrow-mesenchymal stem cells. Three independent samples per group were cryopreserved in liquid nitrogen for 3 months with two different formulations based on inactivated human platelet lysate and compared with a standard solution. **A** Cell viability and **B** recovery post-thaw, **C** recovery at 24-h culture post-thaw, **D** flow cytometry analysis before and after cryopreservation. **E** Immunomodulatory capacity of cryopreserved BM-MSCs measured as the percentage of non-proliferative T-cells relative to the activated peripheral blood mononuclear cell (PBMC) population. **F** Representative carboxyfuorescein succinimidyl ester dye (CFSE) histograms of activated PBMCs both in co-culture with fresh BM-MSCs or with thawed BM-MSCs that were cryopreserved in each solution. **G** Cell stability analysis measured at different time points. Data are shown as mean ± SEM. One-way ANOVA: differences between standard and ihPL-based solutions *P < 0.05; **P < 0.01; ***P < 0.001; differences between CeA and CeB ^#^*P* < 0.05

BM-MSCs are used in multiple clinical applications, including skeletal tissue repair [23] and in treatments for neural disease [24], SARS-CoV-2 infection [25] and liver [26] and pulmonary fibrosis [27], among others. We first tested whether CeA and CeB influenced BM-MSC viability, recovery or immunomodulatory capacity after a 3-month cryopreservation period. Results revealed no significant differences in cell viability between solution CeA and the standard solution (4.5% HSA + 10% DMSO) (one-way ANOVA: *F*_(2,6)_ = 22.09, *P* < 0.01; Tukey’s test: *P* ≤ 0.05; Fig. 3A), with both maintaining viability at ~ 89%. Also, no significant differences were found between the three solutions for cell recovery post-thaw (one-way ANOVA: *P* > 0.05*,* Fig. 3B). Notably, after 24 h of culture, cells cryopreserved with CeA showed a significantly higher recovery than cells cryopreserved with CeB or the standard (one-way ANOVA: *F*_(2,6)_ = 5.148, *P* < 0.05; Tukey’s test: *P* ≤ 0.05; Fig. 3C).

Flow cytometry analysis revealed the similar expression of surface markers CD14, CD34, CD45 and CD73 between cryopreserved and fresh cells. However, we observed a significant loss of CD105 expression in cryopreserved cells after thawing (two-way ANOVA: cryopreservation × solution interaction: *F*_(2,12)_ = 6.995, *P* < 0.01*;* Tukey’s test: *P* ≤ 0.05; Fig. 3D).

No significant differences in immunomodulatory properties were found between the three cryoprotective solutions (*F*_(3,8)_ = 1.365, *P* > 0.05), although BM-MSCs that had been cryopreserved with CeA and CeB had a greater immunosuppressive effect on T-cell proliferation than the standard solution (Fig. 3E, F). Finally, we measured cell stability (viability) of BM-MSCs in the three cryoprotective solutions at 2 h after thawing. Analysis revealed time (*F*_(2,18)_ = 21.01, *P* < 0.0001) and solution (*F*_(2,18)_ = 55.29, *P* < 0.0001) effects between the tested solutions and post hoc analysis showed that BM-MSCs cryopreserved with CeB had a progressively reduced viability approaching the threshold established by the US Food and Drug administration (FDA) (~ 70%) [28] at 2 h post-thaw compared with CeA and the control (Fig. 3G). Overall, the results suggest that CeA is a good candidate to optimize BM-MSC cryopreservation.

#### CeA maintains fibroblast viability > 80% and ensures a good recovery post-thaw and after 24-h culture

**Fig. 4:**
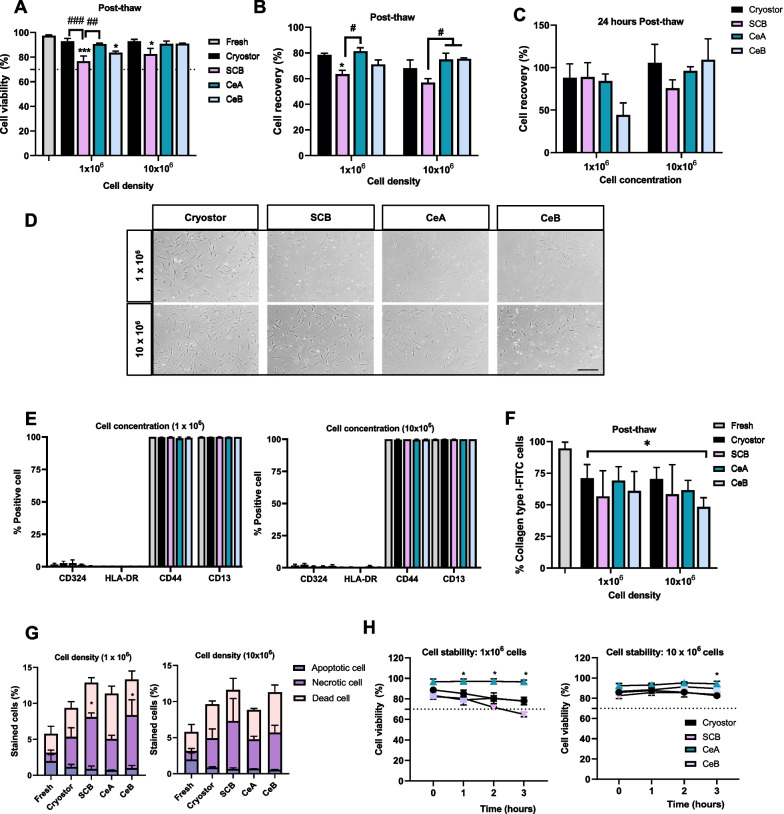
Analysis of cryopreserved dermal fibroblasts. Three independent samples per group were cryopreserved at two cell densities in liquid nitrogen for 9 months in two different formulations based on inactivated human platelet lysate and compared to the commercial products Cryostor® CS10 and STEM-CELLBANKER DMSO FREE-GMP Grade® (SCB). **A** Cell viability and **B** recovery post-thaw. **C** Recovery and **D** phase-contrast images of cells after 24-h culture (scale bar: 100 µm). **E** Flow cytometry-based immunophenotype of fresh and thawed cells cryopreserved at 10^6^ cells/ml (left) and 10 × 10^6^ cells/ml (right). **F** Flow cytometry assessment of collagen type I expression. **G** Flow cytometry analysis of apoptosis at 10^6^ cells/ml (left) and 10 × 10^6^ cells/ml (right) cryopreservation densities. **H** Stability analysis of thawed cells kept in cryopreservation solutions at 10^6^ cells/ml (left) and 10 × 10^6^ cells/ml (right) densities. Data are shown as mean ± SEM. Two-way ANOVA: differences between fresh cell values *P < 0.05; ***P < 0.001; differences between solutions ^#^*P* < 0.05; ^##^*P* < 0.01; ^###^*P* < 0.001

We next tested cryopreserved FBs which can be used to treat dermal diseases [29] and are the most commonly used cell type for induced pluripotent cell generation [30–32], with numerous applications when derived to other cell types [33]. Vials of 1 and 10 × 10^6^ cells/ml of FBs were cryopreserved for 9 months to assess the impact of cell density on cryopreservation. Results revealed an effect of solution on cell viability post-thaw (*F*_(4,20)_ = 17.09, *P* < 0.0001; Fig. 4A), although the viability of cells cryopreserved with CeA was not significantly different to that of fresh cells.

In terms of cell recovery, the ihPL-based solutions gave similar results as those of the CryoStor® CS10 standard, independently of cell density (Tukey’s test: *P* > 0.05, Fig. 4B). Furthermore, we found no significant differences in cell recovery between the cryoprotective solutions after a 24-h culture period (Fig. 4C, D).

Cryopreservation with CeA and CeB did not affect the cell phenotype, as all cells expressed equal levels of the characteristic fibroblast markers CD44 and CD13 at both cell densities in all solutions after thawing (Fig. 4E left and right). By contrast, we observed changes in the expression of collagen type I in cells cryopreserved at both densities relative to fresh cells (two-way ANOVA: solution effect: *F*_(4,19)_ = 3.975, *P* < 0.05; Tukey’s test: *P* > 0.05; Fig. 4F).

Analysis of apoptosis indicated that the annexin V FITC^+^/IP^−^ apoptotic cell population was unchanged by CeA or CeB, independently of cell density (one-way ANOVA: *P* > 0.05, Fig. 4G left and right). However, FBs cryopreserved with SCB and CeB at 1 × 10^6^ cells/ml showed a significantly higher Annexin V FITC^+^/IP^+^ necrotic cell population than fresh FBs (one-way ANOVA: *F*_(4,10)_ = 4.798, *P* < 0.05, Tukey’s test: *P* ≤ 0.05; Fig. 4G left). No significant differences were found between the cryoprotective solutions for the Annexin V FITC^−^/IP^+^ dead cell population at either cell density (one-way ANOVA: *P* > 0.05, Fig. 4G left and right and Additional file 5A and B).

We next analyzed cell stability in terms of viability. Cells in CeA had the greatest cell viability values (~ 95%) independently of cell density (two-way ANOVA: solution effect: *F*_(3,28)_ = 31.82, *P* < 0.0001; time effect: *F*_(3,28)_ = 5.084, *P* < 0.01; Tukey’s test: *P* ≤ 0.05; Fig. 4H left and right). CeB maintained cell viability at ~ 85% when cryopreserved at the highest cell density (Fig. 4H right).

We then explored for potential relationships of the components of the solutions and functional parameters of cells after thawing. Total protein and albumin concentrations positively correlated with cell viability, recovery post-thaw and 24-h culture (Pearson’s correlation: *P* < 0.01, Additional file 6) at 1 × 10^6^ cells/ml. We also found significant differences in cryopreservation at a high cell density, albeit with a weak relationship (*r* near to 0). Additionally, we found that the high concentrations of total protein and albumin in the ihPL-based solutions correlated with less necrotic cells immediately after thawing (Pearson’s correlation: *P* < 0.01, Additional file 6). By contrast, no correlation was found between IgG concentration and other measured parameters, indicating that it likely does not interfere with cell viability or recovery (Additional file 6). Overall, we conclude that CeA is the best candidate to optimize patient-intended FB cryopreservation.

#### Solution CeA maintains NSC viability at 91% with a good performance on recovery post-thaw and doubling time

**Fig. 5:**
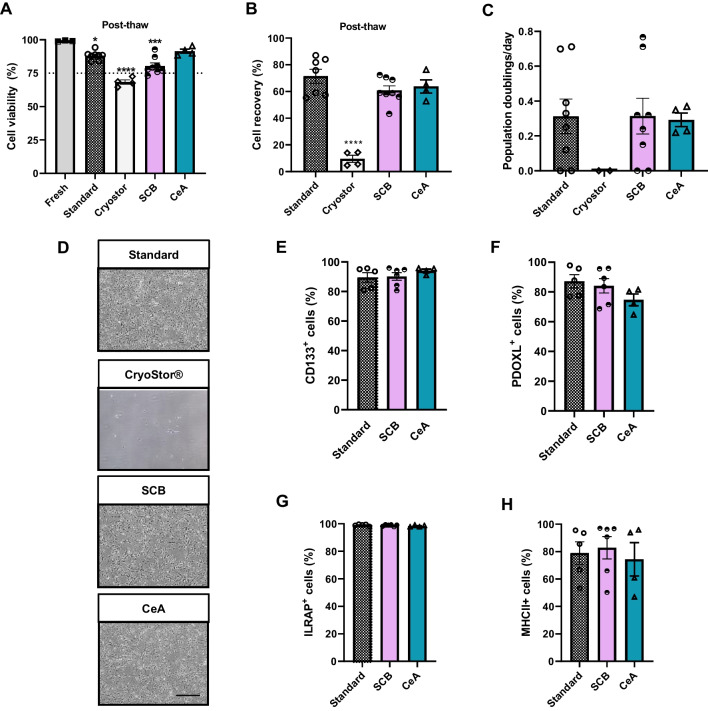
Analysis of cryopreserved neural stem cells. Two or three independent samples per group were cryopreserved in liquid nitrogen for 3 months with the inactivated human platelet lysate cryopreservation solution CeA, and compared to the standard solution (FBS + 10%Me_2_SO) and to the commercial products CryoStor® CS10 and STEM-CELLBANKER® DMSO FREE-GMP Grade (SCB). **A** Cell viability and **B** recovery post-thaw. **C** Representative phase-contrast microphotographs of cells one passage after thawing (scale bar: 100 µm). **D** Cell proliferation (population doublings). Representative histograms of the quantification of surface marker expression: **E** CD133, **F** PODXL, **G** ILRAP and **H** MHCII in thawed cells. The results are presented as mean ± SEM. One-way ANOVA: differences between fresh and cryopreserved cell values/between standard and cryopreservation solutions under assessment: *P < 0.05; ***P < 0.001; ****P < 0.0001

For the analysis of NSCs, we assessed only CeA for optimization, as it provided the best results when tested in the other cell types. We cryopreserved NSCs for a 3-month period with CeA, SCB and the standard solution (Table 2). Results showed that cell viability decreased significantly after cryopreservation with all solutions when compared with fresh cells, with the exception of cells cryopreserved in CeA (one-way ANOVA: *F*_(4,22)_ = 26.91, *P* < 0.0001; Tukey’s test: *P* ≤ 0.05, Fig. 5A), which gave cell viability values of ~ 91% immediately after thawing. Of note, NSCs cryopreserved with CryoSytor® CS10 lost their adherence capacity with a poorer recovery post-thaw or population doublings/day values, which were significantly different to those of the other solutions (one-way ANOVA: *F*_(3,20)_ = 33.72, *P* < 0.0001; Tukey’s test: *P* ≤ 0.05, Fig. 5B, C). No significant differences were found for proliferation ability after thawing (Fig. 5D).

Analysis of the post-thaw phenotype by flow cytometry revealed that the expression of surface markers typical for ventral NSCs (CD133, PDOXL, ILRAP and MHCII) [12] was similar in cryopreserved cells with ihPL-based solutions and with the standard cryopreservation solution (Fig. 5E–H). Cells cryopreserved with CryoSytor® CS10 did not yield a sufficient number for marker expression assessment. Taken together, the results suggest that solution CeA is a good candidate to optimize NSC cryopreservation.

#### Cryoprotective solutions preserve their stability for 18 months

We analyzed the stability of cryoprotective solutions by measuring pH, total protein, albumin and IgG levels to test long-term storage at − 20 °C. Solutions allocated for bioengineered tissues maintained their properties after a 3-month storage (Additional file 7).

**Fig. 6:**
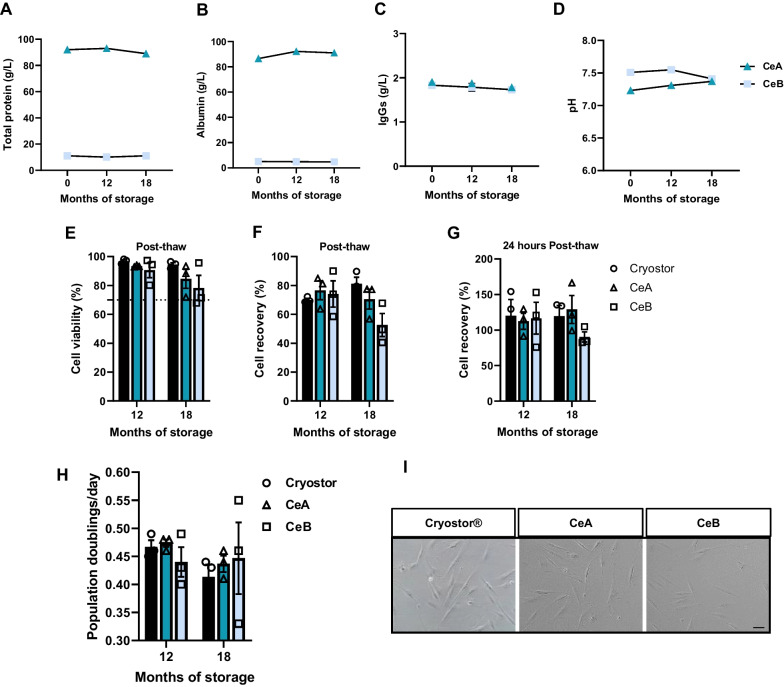
Quantification of components and stability analysis of inactivated human platelet lysate-based solutions for cell cryopreservation. Cryopreservation properties of stored solutions were assessed after long-term storage of 12 and 18 months at − 20 °C. Quantification of components: **A** Dot graph representation of total protein, **B** albumin and **C** immunoglobulin G (IgGs) quantification and **D** pH measurement. Data are shown for one batch per solution. Stored solutions were tested to check whether they maintained their cryopreservation properties. Fibroblasts were cryopreserved and analyzed immediately after thawing: **E** cell viability and **F** recovery measurement. **G** Bar graph representation of cell recovery after 24-h cell culture. **H** Cell proliferation (population doubling). **I** representative phase-contrast microphotographs of cells one passage after thawing (scale bar: 50 µm). Data are shown as mean ± SEM

Solutions allocated for cells preserved their stability for 18 months (Fig. 6A–D). We further tested their cryopreservation stability by cryopreserving FBs with solutions that had been stored at − 20 °C for 12 and 18 months, finding no significant differences in cell viability, post-thaw recovery and 24-h culture when compared with CryoStor® CS10 (Fig. 6E–G). Similarly, no differences were observed for doubling time (Fig. 6H, I). These findings allow us to establish a preliminary expiry date of the product at 18 months from manufacturing.

## Discussion

The effective and efficient cryopreservation of ATMPs is essential for their safe and efficient administration to patients. We show that the cryopreservation solutions proposed for bioengineered tissues (Ti1–5) preserve the viability and functionality of cells embedded in NFAHs, with results similar to those of fresh cells. Cryopreservation per se influenced matrix integrity, but solutions Ti2–Ti5 preserved matrix integrity similar to the standard solution. Solutions Ti4 and Ti5 appeared as the best candidates for cryopreservation of tissues aimed for clinical use, as they have the advantage of being manufactured with ihPL, thus circumventing potential infection risks. The biomechanical properties of the cryopreserved NFAHs were examined to question whether the tested solutions maintained the mechanical properties of the tissue. Results showed that cryopreservation weakened the biomechanical properties of NFAHs, irrespective of the cryoprotective solution used, when compared with fresh tissue, which is consistent with previous studies [13, 14]. We initially set a 7-day cryopreservation but the assessment of cryopreservation for longer periods should be further investigated. Concerning the stability of the NFAHs, future studies should include not only cell viability but also a tissue integrity assessment at different time points (e.g., 1, 2 and 3 h after thawing) by performing a structural analysis and biomechanical properties study. Nonetheless, cell stability results (Fig. 2G, H) show that cells within the scaffold maintain their viability in Solutions Ti4 and Ti5 for at least 3 h after thawing. Given that scaffolds are support structures designed to facilitate cellular growth and proliferation [34], the fact that cell viability remains in such good conditions along this period is also indicative that the scaffold also remains in a good status. Even though, it is important to remark that the stability assessment of NFAHs recreates a worst case scenario where a complication occurs at the operating room that delayed transplant. Otherwise, the tissue would be transplanted immediately after thawing.”

Nevertheless, the applicability of cryopreserved NFAHs must be further examined, as their clinical use may not entail specific biomechanical stress. Our formulations should be tested with other types of bioengineered tissue with different physical properties (e.g., thickness, elasticity, etc.) and containing other cell types. Some studies have reported the successful cryopreservation of 3D-bioprinted osteoblast constructs and beta tricalcium phosphate scaffolds infiltrated with encapsulated osteoblasts [35]. However, although the authors considered eliminating the use of animal components such as FBS, the cryopreservation solution contained 20% DMSO, which may cause adverse reactions in patients. Another study [36] reported the cryopreservation of carboxymethyl cellulose cryogels containing liver hepatocytes with 20% FBS and 5% DMSO. Although the DMSO concentration used was low, the presence of FBS makes this solution inappropriate for human use. The bioengineered tissues studies in the aforementioned reports could be candidates for testing our xeno-free low-DMSO solutions.

Regarding cell cryopreservation, we tested two ihPL-based solutions (CeA and CeB). Solution CeA maintained BM-MSC viability, had no effect on cell recovery or growth and preserved functionality and immunomodulatory properties. Contrastingly, cells cryopreserved with CeB showed significantly reduced viability. Nevertheless, the mean viability values were maintained at ~ 84%, which is 14% higher than the limit established by the FDA [28]. Also, both solutions sustained a stable cell phenotype after cryopreservation, with the exception of a significant loss in CD105 expression. Importantly, BM-MSCs cryopreserved with CeA and CeB had a higher immunosuppressive potency than cells cryopreserved in the standard solution. In this regard, several studies have reported that cryopreservation has a negative impact on the immunomodulatory properties of MSCs when compared with freshly harvested cells [37–39], and Pham et al*.* reported the existence of a subpopulation of CD105- cells with strong immunomodulation properties [40]. These findings suggest that the evident loss of CD105 expression might be related to the immunomodulation capacity retention of our solutions by aiding the preservation of a CD105-low MSC subpopulation, which may be susceptible to cryopreservation.

All BM-MSCs thawed in the novel cryopreservation solutions maintained > 70% viability for two hours. This was done to establish a time-frame in which cells can be infused into patients in the event that cells cannot be maintained frozen before treatment. Solution CeB induced a more rapid decrease in cell viability, and so solution CeA would be a better candidate for clinical applications.

In the case of FBs, cells were cryopreserved in liquid N_2_ during 9 months and further tested for possible effects of cell density against two commercially available cryopreservation solutions commonly used in GMP laboratories: CryoStor® CS10, the standard cryopreservation solution in our laboratory, and SCB, chosen because of its DMSO-free formulation. Furthermore, since the standard cryopreservation density ranges between 1–10 × 10^6^ cells/ml [41, 42], we established these limiting values to test for cell density-related effects. Our solutions allowed good cell recovery post-thaw, similar to CryoStor® CS10, independently of cell density. In the same line as our results, the two aforementioned studies reported no effect of cell density on post-thawing cell quality [41], or found that concentrations higher than typical gave improved post-thaw viability and metabolic activity [42]. Solution CeA was better than CeB to retain cell viability, recovery post-thaw and recovery after 24-h culture, regardless of cell density, but all CPAs solutions triggered changes in the expression of collagen type I. Despite the scarcity of data in this regard, Quintana and colleagues [43] also observed reduced amounts of collagen in fibroblasts from human pulmonary and aortic valves after cryopreservation when compared with fresh valves. Nevertheless, for future studies, cryopreserved cells should be assessed for collagen expression recovery after, at least, 7 days in culture. Finally, analysis of apoptosis revealed that CeA and CeB did not significantly affect the apoptotic FB population at either density, although solution CeA appeared to work better.

Given the current development of ATMPs, there is a need for surgical rooms to have an infrastructure enabling on-site cell processing and graft implantation [44]. Currently, cell/tissue transplantation is performed under conditions that do not allow for the maintenance of specific ATMPs at their required temperatures or for thawing. With this reasoning, and the other multiple events could occur in operating suites that might delay the implantation time, we assessed the viability of thawed FBs in our cryopreservation solutions. We observed that solution CeA showed an outstanding performance in maintaining cell viability at ~ 95% for 3 h regardless of cell density. For all these reasons, we propose solution CeA as the best candidate for FB cryopreservation to treat patients.

Interestingly, our finding of a correlation between both total protein and albumin concentration with cell viability recovery post-thaw and 24-h culture at low cryopreservation density confirm the protective action that albumin exerts at cryopreservation [45].

NSC cryopreservation for clinical use is being tested [46], but an optimal cryoprotective solution has not yet been found. We examined whether CeA could maintain NSC viability and phenotype after a 3-month period. Our solution gave values of ~ 91% cell viability immediately after thawing, showing a better performance than the control solutions. Although cell recovery was not as efficient as with the standard solution (FBS, 10% DMSO), our solution contains a lower DMSO concentration, making it more appealing in a translational context. Importantly, cells cryopreserved in CeA showed similar population doublings per day as those cryopreserved in SCB and the standard solution, but not in CryoSytor® CS10. Marker expression after cryopreservation was similar between all solutions.

To our knowledge, no studies have assessed the stability of hPL-based cryopreservation solutions. We found both CeA and CeB maintained their properties during 18 months of storage at − 20 °C. Additionally, both solutions but especially CeA achieved good results for cryopreserving FBs, allowing us to establish a preliminary expiry date of the product at 18 months from manufacturing.

Our ihPL-based solutions are safe and efficient candidates to cryopreserve ATMPs, offering a xeno-free and low-DMSO alternative to commercially available solutions. Nevertheless, several other tissues should be examined in future studies. Moreover, cell potency must be further investigated in future studies in a case-based analysis, according not only to the specific cell type but also to the precise pathology to be treated. Also, the DMSO concentration in both bioengineered tissues and cells cryopreserved in ihPL-based solutions should be determined before and after performing a PBS wash, as proposed by Herrero-Gómez et al. [36], to evaluate whether this step could be advantageous in diminishing DMSO content and, thus, its toxicity. Other strategies for DMSO dilution could be applied, as previously reported [18].

## Conclusions

Our ihPL-based Ti5 and CeA solutions are safe and efficient candidates to cryopreserve ATMPs, offering a xeno-free and low-DMSO alternative to commercially available solutions. Additionally, cryopreserved cells are stable in an operating room scenario that does not have equipment specific for ATMP maintenance, where multiple events can occur delaying their grafting/infusion.

## Supplementary Information

**Additional file 1.** Freezing programs. Description of data: table compiling the controlled rate freezer programs used to cryopreserve cellularized nanostructured fibrin agarose hydrogels (NFAHs) and bone marrow-derived mesenchymal stromal cells (BM-MSCs).

**Additional file 2.** List of antibodies used for immunofluorescence assays of cellularized nanostructured fibrin agarose hydrogels (NFAHs). Description: table compiling the antibodies used for immunofluorescence assays.

**Additional file 3.** List of antibodies used for flow cytometry analysis. Description: table compiling the antibodies used for flow cytometry analysis.

**Additional file 4.** Graph bar representation of measured thawing time of cellularized nanostructured fibrin agarose hydrogels (NFAHs) cryopreserved with different solutions. Description: **A** NFAHs cryopreserved with solution Ti3 took significantly longer to thaw (one-way ANOVA: F_(6,24)_ = 7.222, *P* < 0.0001, Tukey’s test: *P* < 0.05). **B** Graph shows no correlation between thawing time and interfibrillar space increase (Pearson’s correlation: *P* > 0.05).

**Additional file 5.** Forward scatter (cell size) versus side scatter (cell complexity) dot plot analysis of fibroblasts (FBs) after cryopreservation with all proposed cryopreservation solutions. Description: There were no differences observed between FBs that were cryopreserved at low density: **A** 1 × 10^6^ cells/ml or **B** high density: 10 × 10^6^ cells/ml.

**Additional file 6.** Pearson’s comparison between cryoprotective solutions components and functionality parameters in human fibroblast cryopreservation. Description: Table compiling Pearson’s correlation coefficients (*r*) and corresponding *P* values when cryoprotective solutions components and functionality parameters in human fibroblast cryopreservation are compared.

**Additional file 7.** Total protein, albumin, IgG and pH measurements in cryoprotective solutions allocated for cryopreservation of cellularized nanostructured fibrin agarose hydrogels (NFAHs). Description: table compiling total protein, albumin, IgG and pH measurements in cryoprotective solutions allocated for NFAH cryopreservation.

## Data Availability

The data that support the findings of this study are available from MD Bioproducts GmbH but restrictions apply to the availability of these data and so are not publicly available. Data are, however, available from the authors upon reasonable request and with the permission of MD Bioproducts GmbH.

## Abbreviations

ATMPs: Advanced therapy medicinal products
BM-MSCs: Bone marrow-derived mesenchymal stromal cells
FBs: Dermal fibroblasts
NSCs: Neural stem cells
iPSCs: Induced pluripotent cells
CPA: Cryoprotective agent
hPL: Human platelet lysate
FBS: Fetal bovine serum
DMSO: Dimethyl sulfoxide
ihPL: Inactivated human platelet lysate
NFAH: Cellularized nanostructured fibrin agarose hydrogel
RT: Room temperature
PBMCs: Peripheral blood mononuclear cells
IgG: Immunoglobulin G
HIER: Heat-induced epitope retrieval
