# Supplementary material for: Bioengineered tissue and cell therapy products are efficiently cryopreserved with pathogen-inactivated human platelet lysate-based solutions
Source: Stem Cell Res Ther. 2023 Apr 7;14:69. doi: 10.1186/s13287-023-03300-z (PMC10079488; doi:10.1186/s13287-023-03300-z)
Supplement: Supplementary file 1 — Additional file 1. Freezing programs. Description of data: table compiling the controlled rate freezer programs used to cryopreserve cellularized nanostructured fibrin agarose hydrogels (NFAHs) and bone marrow-derived mesenchymal stromal cells (BM-MSCs). [file 13287_2023_3300_MOESM1_ESM.pdf]

Table. Additional file 1

Additional file 1. Freezing programs

Advanced Therapy Products	Controlled rate freezer	GMP Laboratories
NFAH	30 min on ice<br>24ºC at -20ºC<br>48ºC at -80ºC<br>Transfer to -196ºC	UPRC Seville
BM-MSCs	Wait at 4ºC<br>Ramp 0.1ºC min^{-1} until Chamber = 2ºC<br>Ramp 1ºC min^{-1} until Chamber = -10ºC<br>Ramp 71ºC min^{-1} until Chamber = -60ºC*<br>Ramp 25ºC min^{-1} until Chamber = -20ºC<br>Ramp 0.2ºC min^{-1} until Chamber = -21ºC<br>Ramp 1.2ºC min^{-1} until Chamber = 50ºC<br>Ramp 7ºC min^{-1} until Chamber = -120ºC<br>Transfer to -196ºC	HURS Cordoba

*This step induces extracellular ice nucleation near the freezing point of the cryoprotective solution (-10ºC)

NFAH: cellularized nanostructured fibrin agarose hydrogel; BM-MSCs: bone marrow-derived mesenchymal stromal cells; UPRC: Unidad de Producción y Reprogramación Celular; HURS: Hospital Reina Sofía
