# Supplementary material for: Bioengineered tissue and cell therapy products are efficiently cryopreserved with pathogen-inactivated human platelet lysate-based solutions
Source: Stem Cell Res Ther. 2023 Apr 7;14:69. doi: 10.1186/s13287-023-03300-z (PMC10079488; doi:10.1186/s13287-023-03300-z)
Supplement: Supplementary file 2 — Additional file 2. List of antibodies used for immunofluorescence assays of cellularized nanostructured fibrin agarose hydrogels (NFAHs). Description: table compiling the antibodies used for immunofluorescence assays. [file 13287_2023_3300_MOESM2_ESM.pdf]

Table S2. List of antibodies used for immunofluorescence assays of cellularized nanostructured fibrin agarose hydrogels

Primary antibodies	Dilution and Supplier
rabbit anti-collagen type I	1:500; Abcam
mouse anti-vimentin	1:1000; Abcam
Alexa-Fluor™ 647 Phalloidin	1: 100; Invitrogen

Secondary antibodies	Dilution and Supplier
Alexa Fluor® 488 donkey anti-rabbit IgG (H+L)	1:250; Invitrogen
Alexa Fluor® 594 plus donkey anti-mouse IgG (H+L)	1:250; Invitrogen
