# Supplementary material for: Bioengineered tissue and cell therapy products are efficiently cryopreserved with pathogen-inactivated human platelet lysate-based solutions
Source: Stem Cell Res Ther. 2023 Apr 7;14:69. doi: 10.1186/s13287-023-03300-z (PMC10079488; doi:10.1186/s13287-023-03300-z)
Supplement: Supplementary file 3 — Additional file 3. List of antibodies used for flow cytometry analysis. Description: table compiling the antibodies used for flow cytometry analysis. [file 13287_2023_3300_MOESM3_ESM.pdf]

Table. Additional file 3

Additional file 3. List of antibodies used for flow cytometry analysis

Cell type	Fluorochrome-conjugated antibodies	Dilution and Supplier
BM-MSCs	CD14-PE	1:50; Miltenyi Biotec
	CD34-APC	1:50; Biolegend
	CD45-FITC	1:50 Miltenyi Biotec
	CD73-PE	1:50 Miltenyi Biotec
	CD90-FITC	1:50 Miltenyi Biotec
	CD105-APC	1:50 Miltenyi Biotec
FBs	CD44	1:50; Miltenyi Biotec
	CD13	1:50; Biolegend
	CD324	1:50 Miltenyi Biotec
	HLA-DR	1:50 Miltenyi Biotec
PBMCs	CFSE	5 µM Sigma Aldrich
	CD3-APC	1:50 Miltenyi Biotec
	CD4-PE	1:50 Miltenyi Biotec
	CD8-APC Vio770	1:50 Miltenyi Biotec
NSCs	CD133-PE	1:11 Miltenyi Biotec
	Podocalixyn-PE	1:11 R&D
	IL1RAP-AF488	1:11 R&D
	MHC-II-APC	1:11 Miltenyi Biotec

BM-MSCs: bone marrow-derived mesenchymal stromal cells; FBs: human dermal fibroblasts; PBMCs: peripheral blood mononuclear cells; NSCs: neural stem cells
