# Supplementary figures and images for: Bioengineered tissue and cell therapy products are efficiently cryopreserved with pathogen-inactivated human platelet lysate-based solutions
Source: Stem Cell Res Ther. 2023 Apr 7;14:69. doi: 10.1186/s13287-023-03300-z (PMC10079488; doi:10.1186/s13287-023-03300-z)

Additional file 4

A

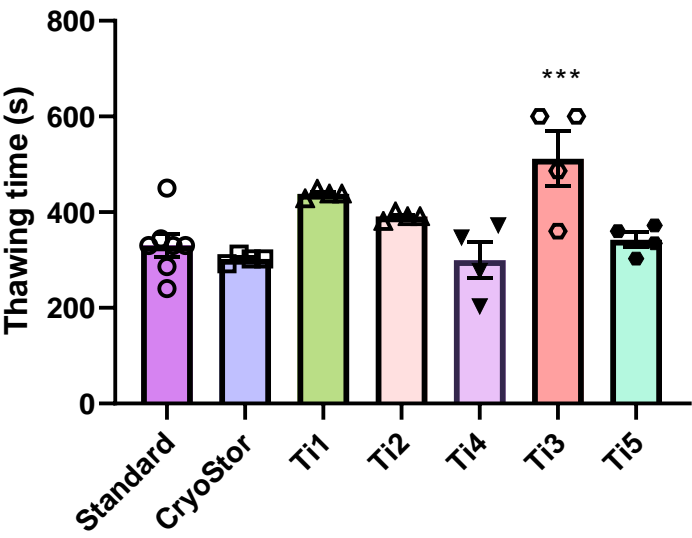

B

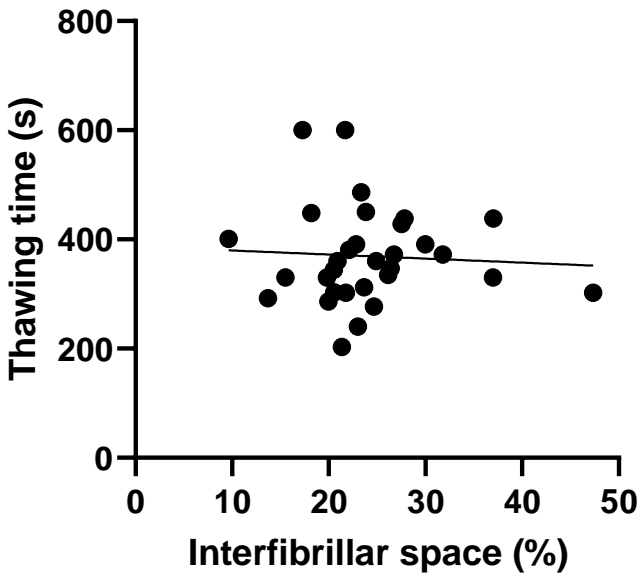

Supplement: Supplementary file 4 — Additional file 4. Graph bar representation of measured thawing time of cellularized nanostructured fibrin agarose hydrogels (NFAHs) cryopreserved with different solutions. Description: A NFAHs cryopreserved with solution Ti3 took significantly longer to thaw (one-way ANOVA: F(6,24) = 7.222, P < 0.0001, Tukey’s test: P < 0.05). B Graph shows no correlation between thawing time and interfibrillar space increase (Pearson’s correlation: P > 0.05). [file 13287_2023_3300_MOESM4_ESM.pdf]

Additional file 5

A

Cell density: 1x10<sup>6</sup> cells/ml

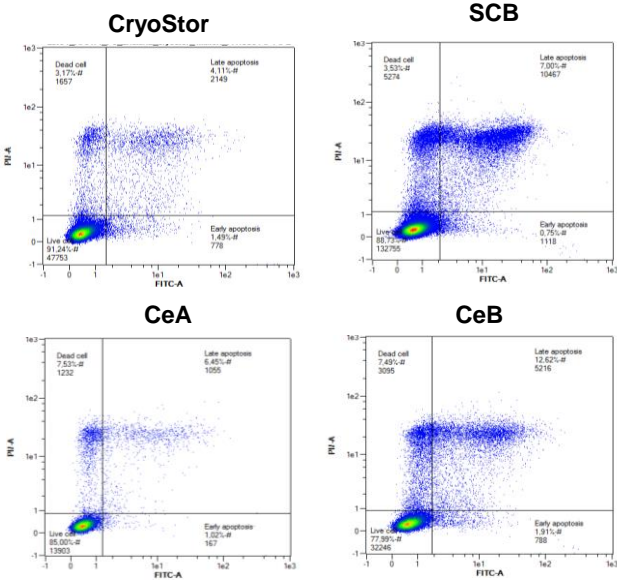

B

Cell density: 10x10<sup>6</sup> cells/ml

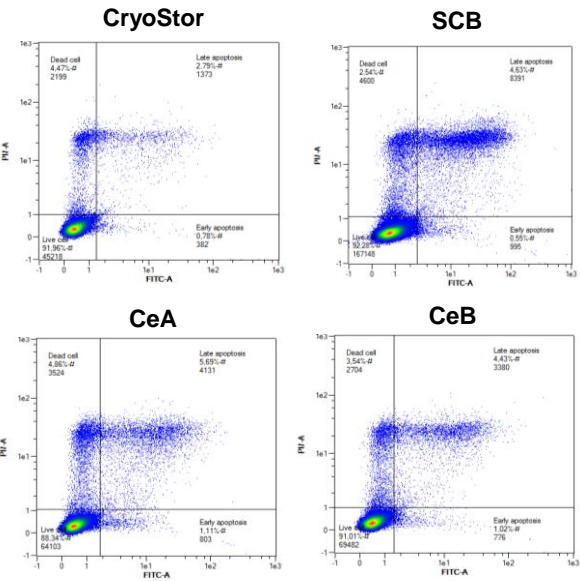

Supplement: Supplementary file 5 — Additional file 5. Forward scatter (cell size) versus side scatter (cell complexity) dot plot analysis of fibroblasts (FBs) after cryopreservation with all proposed cryopreservation solutions. Description: There were no differences observed between FBs that were cryopreserved at low density: A 1 × 10^6^ cells/ml or B high density: 10 × 106 cells/ml. [file 13287_2023_3300_MOESM5_ESM.pdf]
