# Supplementary material for: Bioengineered tissue and cell therapy products are efficiently cryopreserved with pathogen-inactivated human platelet lysate-based solutions
Source: Stem Cell Res Ther. 2023 Apr 7;14:69. doi: 10.1186/s13287-023-03300-z (PMC10079488; doi:10.1186/s13287-023-03300-z)
Supplement: Supplementary file 6 — Additional file 6. Pearson’s comparison between cryoprotective solutions components and functionality parameters in human fibroblast cryopreservation. Description: Table compiling Pearson’s correlation coefficients (r) and corresponding P values when cryoprotective solutions components and functionality parameters in human fibroblast cryopreservation are compared. [file 13287_2023_3300_MOESM6_ESM.pdf]

Table. Additional file 6

Additional file 6. Pearson's comparison between cryoprotective solutions components and functionality parameters in human fibroblast cryopreservation

Pearson's Correlation	Total Protein		Albumin		IgGs	
	10^6 cells/mL density	$10x10^6$ cells/mL density	10^6 cells/mL density	$10x10^6$ cells/mL density	10^6 cells/mL density	$10x10^6$ cells/mL density
Viability post-thaw	0.921<br>0.0001****	0.000<br>0.0001****	0.920<br>0.0001****	0.000<br>0.0001****	0.921<br>0.067	0.000<br>0.197
Recovery post-thaw	0.603<br>0.0001****	0.054<br>0.0001****	0.762<br>0.0001****	0.055<br>0.0001****	0.684<br>0.203	-0.055<br>0.528
Recovery 24 h post-thaw	0.471<br>0.011*	0.044<br>0.0001****	0.471<br>0.008**	0.000<br>0.388	0.471<br>0.415	0.054<br>0.393
Necrotic cell	0.566<br>0.012*	-0.445<br>0.004**	0.566<br>0.01**	0.445<br>0.003**	0.566<br>0.214	0.448<br>0.313

Pearson's correlation coefficients (*r*) are shown above and p-values are shown below. (*) $P < 0.05$; (**) $P < 0.01$; (****) $P < 0.0001$
