# Supplementary material for: Bioengineered tissue and cell therapy products are efficiently cryopreserved with pathogen-inactivated human platelet lysate-based solutions
Source: Stem Cell Res Ther. 2023 Apr 7;14:69. doi: 10.1186/s13287-023-03300-z (PMC10079488; doi:10.1186/s13287-023-03300-z)
Supplement: Supplementary file 7 — Additional file 7. Total protein, albumin, IgG and pH measurements in cryoprotective solutions allocated for cryopreservation of cellularized nanostructured fibrin agarose hydrogels (NFAHs). Description: table compiling total protein, albumin, IgG and pH measurements in cryoprotective solutions allocated for NFAH cryopreservation. [file 13287_2023_3300_MOESM7_ESM.pdf]

Table. Additional file 7

Additional file 7. Total protein, albumin, IgG and pH measurements in cryoprotective solutions allocated for cryopreservation of cellularized nanostructured fibrin agarose hydrogels.

Cryoprotective solutions	Total Protein (g/l)		Albumin (g/l)		IgGs (g/l)		pH	
	t=0	t=3	t=0	t=3	t=0	t=3	t=0	t=3
Solution Ti4	33	36	29,8	29.8	1.47	1.5	7.09	7.06
Solution Ti5	86	91	85.9	84.5	1.50	1.54	7.15	7.21

hPL: human platelet lysate; ihPL: inactivated human platelet lysate; HSA: human serum albumin; IgG: immunoglobulin G
